# Association between social asymmetry and depression in older adults: A phone Call Detail Records analysis

**DOI:** 10.1038/s41598-019-49723-8

**Published:** 2019-09-18

**Authors:** Timothée Aubourg, Jacques Demongeot, Félix Renard, Hervé Provost, Nicolas Vuillerme

**Affiliations:** 1 Univ. Grenoble Alpes, AGEIS, Grenoble, France; 2 Orange Labs, Meylan, France; 3 LabCom Telecom4Health, Univ. Grenoble Alpes & Orange Labs, Grenoble, France; 4 Institut Universitaire de France, Paris, France

**Keywords:** Diagnostic markers, Depression, Translational research, Statistics

## Abstract

Analyzing social interactions on a passive and non-invasive way through the use of phone call detail records (CDRs) is now recognized as a promising approach in health monitoring. However, deeper investigations are required to confirm its relevance in social interaction modeling. Particularly, no clear consensus exists in the use of the *direction* parameter characterizing the directed nature of interactions in CDRs. In the present work, we specifically investigate, in a 26-older-adults population over 12 months, whether and how this parameter could be used in CDRs analysis. We then evaluate its added-value for depression assessment regarding the *Geriatric Depression Scale* score assessed within our population during the study. The results show the existence of three clusters of phone call activity named (1) *proactive*, (2) *interactive*, and (3) r*eactive*. Then, we introduce the notion of asymmetry that synthesizes these activities. We find significant correlations between asymmetry and the depressive state assessed in the older individual. Particularly, (1) *reactive* users are more depressed than the others, and (2) not depressed older adults tend to be *proactive*. Taken together, the present findings suggest the phone’s potential to be used as a social sensor containing relevant health-related insights when the *direction* parameter is considered.

## Introduction

The analysis of phone call detail records (CDRs) is now recognized to represent a promising approach for contributing to the future of mental health research^1^. For the data scientist, it gives the opportunity to model real-time human social activity on a digital way by combining the growing capability of computers with the massive amount of data generated by the daily use of the phone ubiquitous technology^2^. For the health professional, it brings new tools enabling monitoring of numerous traits, as sleep, mood variation and social interaction of his patient to preserve his health and wellness through time^3–5^. Last years, combining the competences of both of these experts has shown a huge potential for enhancing the health care system in various aspects, by improving detection, screening and diagnosis of mental illness, but also by monitoring lifestyle and symptoms on a low-cost and non-invasive way by means of phone-generated passive data^6^. As a consequence, this potential has materialized recently through a rich and expansive literature in the field of mobile health (e.g., see^7,8^ for two recent reviews), so-called *mHealth*, but also in the specific field of CDRs analysis itself. (e.g., see^2,9^ for two recent reviews). On the whole, this scientific literature emphasizes the increasingly potential of phone telecommunication datasets to be harnessed for modeling human activity at both population and individual levels^10–12^, as well as their interest to be used for good purpose, notably in health^13^.

It is interesting to note that despite the great interest raised by such an approach, some aspects of CDRs analysis still require careful further study and discussion. Particularly, we can mention that no clear consensus exists among researchers in the use of the phone call direction parameter, which permits to distinguish outgoing from incoming phone calls in CDRs datasets^9^. Presently, the use or not of this parameter often does depends on *a priori* decisions taken by researchers that are inconsistent regarding the level of investigation of their studies^14–20^. At aggregate-level for instance, we can mention the work led by^14^ in which authors assess the overall population’s phone call circadian activity by analyzing outgoing phone calls only. They exclude incoming ones because these last ones‘ may not depend on the activity pattern of the users‘ (^14^ Page 3). On the contrary, at network-level, both outgoing and incoming phones calls are often considered when leading complex system analyses onto CDRs datasets^15,17^. Nevertheless, in order to figure out stable, reciprocal, or undirected network structures’ properties, the phone call direction parameter is often removed from such studies after the data pre-processing step. As acknowledged by other authors in a recent work, ‘considering direction might convey a more nuanced interpretation of the notion of strength itself’ although ‘it would require introducing an additional hypothesis’ (^16^ Page 228). Interestingly, at individual-level, we note that combining outgoing and incoming data without distinction does also appear in general exploratory studies^18^, whereas those specifically applied for a health purpose tend to distinguish both of them^19,20^. In particular, in *mhealth*, removing the phone call direction parameter from the statistical analysis does not represent a common situation even though, to the best of our knowledge, no investigation specifically addressed the added-value of such a parameter in implementing statistical models. Actually, it must be pointed out that some of these studies in *mhealth* have provided relevant results when analyzing outgoing and incoming phone calls separately^19,20^. Particularly, we can mention the work in^19^ in which the phone activity of 29 patients with bipolar depression, and having moderate to severe levels of depressive and manic symptoms, over a 12 weeks period was analyzed. In this study, authors conclude that the more severe the depressive symptoms (1) the more the patient receives phone calls, and (2) fewer outgoing calls are made. In contrast, the more severe the manic symptoms the more the patient send outgoing text message. Hence, by taking advantage of the direction parameter in CDRs, authors show the existence of relevant insights based on the directed nature of phone calls/text messages regarding the health state of the individual, although this direction is not the primary outcome of the study. Nevertheless, we also have to mention that no additional investigation is made to explicitly quantify the real interest of such a parameter, which exhibits two clear limits in finding.

First, as mentioned in a recent systematic review^7^ about correlations between analyzed passive data and depressive mood symptoms in patients, numerous studies have reported discrepancies, or even contradictory, results when measuring the degree of relation between social interaction data, such as call conversations and text messages, and the depressive symptoms of the individual. Thus, cautious is needed when considering results of^19^. Authors of the corresponding review^7^ stress these different findings and conclude in the need of consistency in analysis. However, we can argue that it also seems plausible that such differences could, logically, underline the existence of different phone-usage habits in humans. All in all, further investigations are needed to investigate the relevance of such potential habits in a *mHealth* context. Second, to the best of our knowledge, as previously done in most *mHealth* studies (e.g. see^7,8^ for two recent reviews on the subject), the present work carried out in^19^ analyses outgoing and incoming data separately without mentioning their intrinsic relation with the concept of action-reaction in social interactions. Consequently, previous articles do not investigate the existence of a balance between activity and reactivity in phone calls and its plausible relation with the individual’s depressive state. In human, this principle of action-reaction between the individual and his environment, called “*enaction*”^21^, is however fundamental for defining, at least in part, the notion of behavior^22^. What is more, in the field of social psychology, there are numerous evidences that different properties of the individual’s social environment, including social interactions, may have a profound effect on the health and well-being of individuals^23–26^. In particular, the notion of ‘social support’ – that refers to “positive interaction or helpful behavior provided to a person in need of support” (^27^ Hupcey Page 2) – may play a crucial role for both physical and mental health issues^26,28^, via notably the loneliness phenomena^29^. This last one can be defined as a negative emotional state corresponding to a misalignment between desired and achieved social interaction patterns^30^. It has been found to be largely negatively correlated with the social support perceived by the individual^29^. In later life, its role may be directly associated with the existence of severe mental health issues as depression^31^. Hence, all in all, analyzing outgoing and incoming social interactions occurring through phone seems a relevant way to address the existence of different phone-usage habits in human, but also to propose a quantification of the phenomena of social support in order to estimate its association with the individual’s depressive state.

In this train of thought, this paper is designed to investigate the interest of the phone call direction parameter in CDRs analysis for an older population. We specifically address two main issues: (1) the existence of a general phone call habit in the older adult regarding the direction of his phone calls, and (2) the existence of a relation between such a habit and the depressive health state of the individual. To this end, we use a 12-successive-month dataset that combines CDRs and geriatric depressive scales (GDS) results of 26 volunteers older than 65 years. On the whole, our results show the existence of three clusters of phone call habits named (1) *proactive*, (2) *interactive*, and (3) *reactive*. Interestingly, by introducing two asymmetry indicators, namely (1) the asymmetry coefficient, and (2) the skewness coefficient, that permit to quantify and to synthesize these three phone call habits, we find a significant correlation between them and the GDS score obtained by the older individual. In particular, we observe that older individuals having a r*eactive* habit obtain GDS results significantly higher than the other ones. In contrast, individuals obtaining a GDS lower than 10 tend to have asymmetry coefficients with positive values. The significance and limitations of this study are discussed and a future research direction is proposed.

## Results

Results presented in this section follow an approach divided into two successive processes, namely (1) we analyze the asymmetry in phone call activity in older adults by comparing outgoing phone calls with incoming phone calls, and (2) we synthesize these results by means of asymmetry indicators to assess the existence of a relation between asymmetry in phone call activity and the depressive health state of the older individual assessed by the GDS.

### Three distinct clusters of phone call activity are observed in older adults

**Figure 1:**
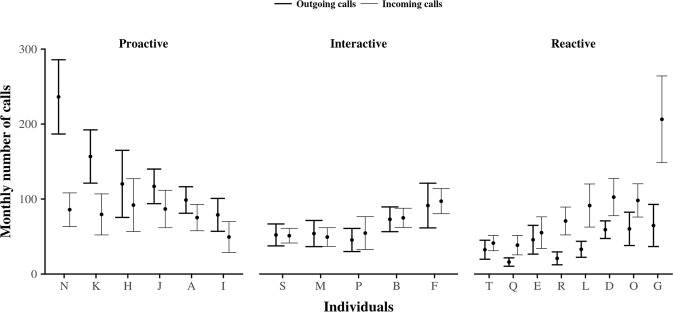
Phone call activity behaviors in older population regarding the monthly number of phone calls and their direction. Bold lines represent outgoing phone calls whereas thin lines represent incoming ones. For each individual, the total average of his monthly number of phone calls is represented by a black dot, whereas the corresponding standard deviation is represented by an error bar. Here, we observe three distinct clusters in older adults: (1) those who phone more than they respond to their incoming phone calls (*proactive* users), (2) those who phone as much as they respond to their incoming phone calls (*interactive* users), and (3) those who phone less than they respond to their incoming phone calls (*reactive* users). Such behaviors emphasize the fact that, behind the phone ubiquitous status, different phone call activity use-cases exist in older adults.

Figure 1 shows the outgoing phone call activity and the incoming phone call activity of each individual calculated by month. By comparing outgoing and incoming phone calls by means of appropriate Wilcoxon comparison tests with a p-value set at 0.05 (see data analysis in Methods section), results figure out the existence of three distinct behaviors in phone call habits, named: (1) *proactive*, (2) *interactive*, and (3) *reactive*.

**Figure 2:**
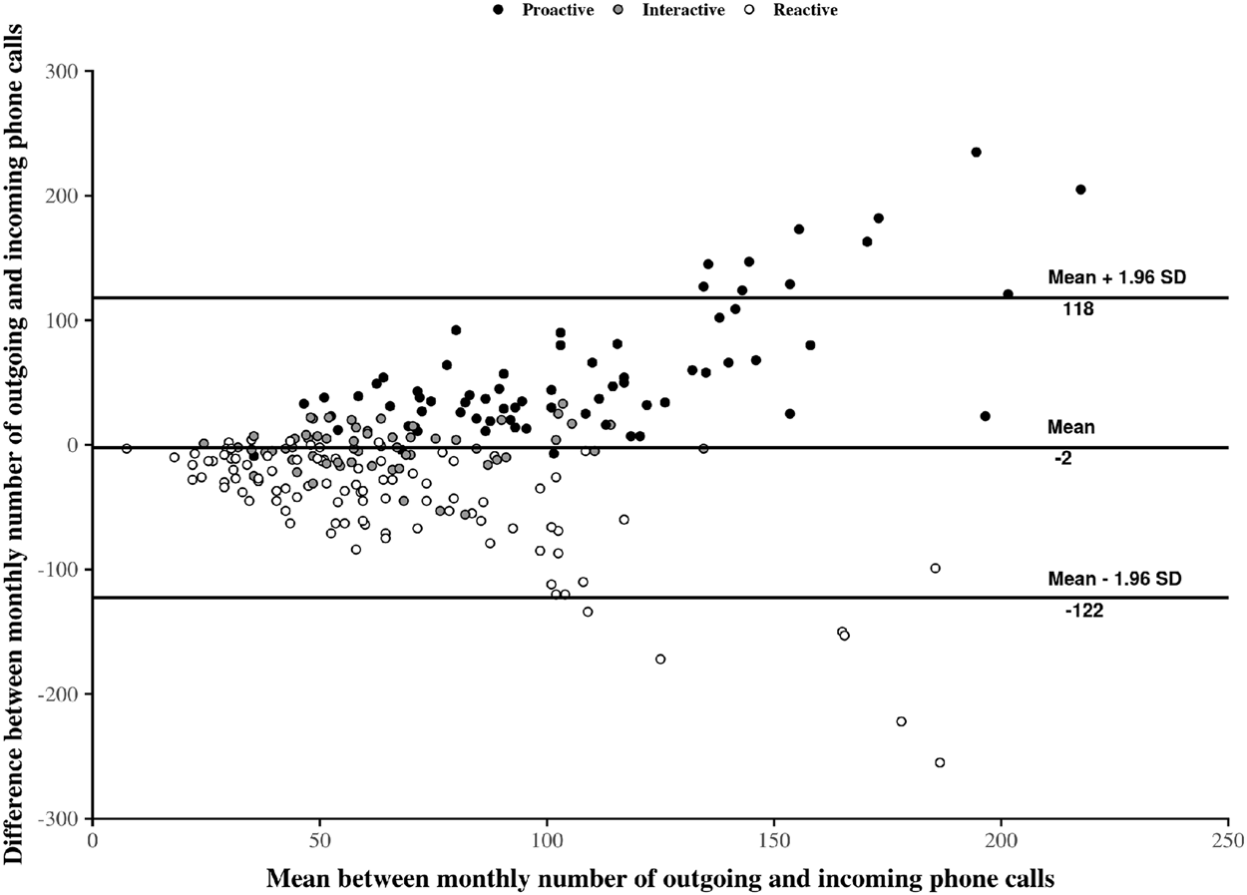
Bland-Altman plot for outgoing phone calls and incoming phone calls. Individuals are represented by dots whose colors are assigned according to the individual’s general phone call activity behavior. On this figure, two points stand out: (1) the general bias of difference between the monthly number of outgoing phone calls and the monthly number of outgoing phone calls is low for the overall population, but (2) when considering each clusters of phone call activity, we observe that only *interactive* users seem to have such a low bias, whereas there is a sharp contrast between *Proactive* users and *Reactive* phone users. In short, there is a low agreement between outgoing phone calls and incoming phone calls depending on the considered cluster of phone call activity. Taken together, these observations emphasize the fact that different behaviors exist in phone call activity in older adults. In particular, depending on these behaviors, there is no evidence in neglecting the phone call activity direction when leading statistical analysis.

Descriptive statistics are summarized in Table 2. Results show that considering the overall population without behavioral distinction among individuals conducts to misleading interpretations. Indeed, regarding the overall population, the monthly average of the outgoing phone calls’ number is close to the number of incoming ones with respectively 77 (SD = 53) versus 79 (SD = 37) monthly phone calls. In contrast, considering users according to their behavioral cluster leads to other interpretations. On the one hand, *proactive* users phone more than they respond to phone calls with respectively 135 (SD = 19) versus 78 (SD = 15) monthly phone calls in average. On the other hand, *reactive* users phone less than they respond to phone calls with respectively 42 (SD = 19) versus 88 (SD = 54) monthly phone calls in average. Only *interactive* users phone as much as they respond to phone calls with respectively 68 (SD = 19) versus 65 (SD = 20) monthly phone calls in average. Then, regarding each of these *proactive*, *interactive*, and *reactive* clusters, correlation analyses show a significant positive relation between the monthly number of outgoing phone calls and the monthly number of incoming phone calls (p-values < 1e-06) with a Pearson’s rho equals to, respectively, 0.53, 0.74, and 0.62. Additionally, the slope and the intercept of the corresponding linear regressions, regarding each cluster, confirm the existence of three distinct behaviors in phone call habits. Last, by assessing the agreement of the relation existing between outgoing and incoming phone calls, Bland-Altman graphical analyses displayed on Fig. 2 confirm the existence of a bias of interpretation when considering only the phone call activity of the overall population without distinction between clusters. This bias is suggested by the shape of the scatter plot, which seems to follow several, different and opposite trends, but also by the wide interval of 95%-limit of agreement (−122–118). Then, considering each cluster independently, a bias represented by a positive mean of differences and by a negative one does also exist for *proactive* and for *reactive* users with a value of respectively 57 and −47. This bias is close to zero (−2) for *interactive* users. Taken together, all these statistical results emphasize the existence of different behaviors in phone call activity in older adults. In particular, two insights stand out: (1) the overall phone call activity of the population is not necessary representative of individuals who show distinct behaviors in phoning, and (2) when considering these behaviors, there is no evidence in neglecting the directed nature of phone calls because of the significant differences existing between outgoing phone calls and incoming phone calls.

### Phone calls’ asymmetry indicators permit to characterize *proactive*, *interactive*, and *reactive* users

To characterize the three clusters described above with simple metrics, we measure the balance in phone call activity in older adults by introducing two asymmetry indicators, named (1) the asymmetry coefficient AC, and (2) the Skewness coefficient SC (see Asymmetry Indicators in Methods).

**Figure 3:**
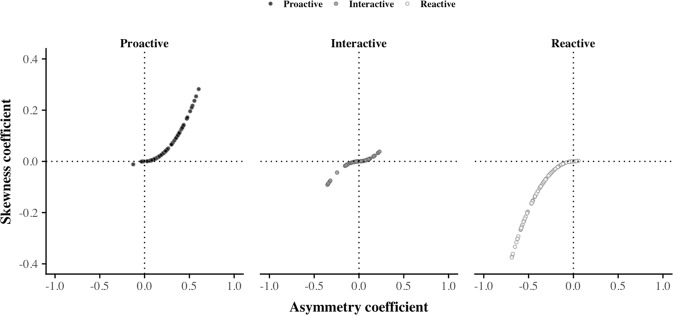
Asymmetry in phone call activity behaviors in older population regarding the outgoing and incoming monthly number of phone calls. For each cluster of phone call activity, the asymmetry coefficient and the information of direction values are calculated for each individual at each month. Their association is then represented by a dot displayed onto the corresponding panel. Here, the dot’s color is assigned according to the individual’s general phone call activity behavior. On this figure, we can observe that while *proactive* and *reactive* users are characterized by a wide range of, respectively, positive and negative values, *interactive* users are characterized by both values oscillating around zeros, but also by a few highly positive and negative ones. Thus, taken together, these observations highlight the existence of variations between *proactive*, *reactive*, and *interactive* users, as well as the asymmetry indicator’ ability in catching their characteristics.

Figure 3 shows the values taken by these asymmetry indicators values, calculated each month of the year for each individual. On the whole, *proactive* and *reactive* users are characterized by a wide range of, respectively, positive and negative values, while *interactive* users are characterized by both values oscillating around zero, but also by a few highly positive and negative ones. By using Kruskall-Wallis comparison tests based on both AC and SC, we then confirm that *proactive*, *interactive*, and *reactive* individuals originate from three different distributions. This result confirms the ability of asymmetry indicators for characterizing each of these three behavioral clusters. In particular, we can mention that, although AC and SC provide similar results, their values do not follow a completely similar distribution. Actually, as illustrated on Fig. 3, SC tends to ‘compress’ values around zero and to dilate the other ones in comparison with AC values. This aspect implies that using SC when characterizing the users’ behavior may conduct to more insist on those with a prominent *proactive* or *reactive* behavior and to less consider the other ones. In comparison, AC more insists on individuals with values around zero.

### Interest of asymmetry indicators for assessing depression in older adults

**Figure 4:**
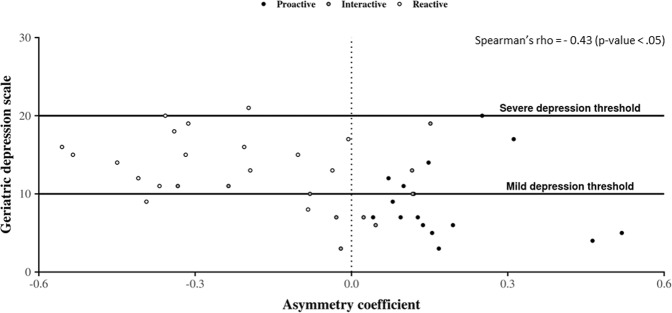
Geriatric Depression Scale values’ distribution according to asymmetry coefficient values. Here, dots correspond to the association between the individual’s GDS value and the individual’s asymmetry coefficient value calculated from CDRs over the 4-week period before the date at which the older adult passed the GDS test. Their colors are assigned according to the individual’s general phone call activity behavior. On this figure, we observe that individuals with a negative asymmetry coefficient tend to obtain a high GDS score corresponding mostly to mild depression. In contrast, individuals obtaining a low GDS score tend to have a positive asymmetry coefficient.

**Figure 5:**
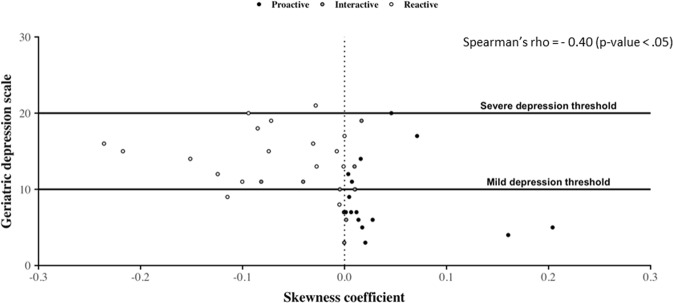
Geriatric Depression Scale values’ distribution according to the skewness coefficient values. Dots correspond to the association between the individual’s GDS value and the individual’s skewness coefficient value calculated from CDRs over the 4-week period before the date at which the older adult passed the GDS test. Their colors are assigned according to the individual’s general phone call activity behavior. On this figure, we observe that, similarly to the asymmetry coefficient, individuals with a negative value of their skewness coefficient tend to obtain a high GDS score corresponding mostly to mild depression. In contrast, individuals obtaining a low GDS score tend to have a positive value of information of direction.

Figures 4 and 5 show the distribution of GDS score in older adults according to their asymmetry indicators. Since individuals were tested regarding their depressive state up to three times during the year of observation, and because the date of each test does not necessarily correspond to the end of a month, we keep in this part only CDRs matching with the 4-weeks period of time before the date corresponding to each health test, for each individual. We then calculate asymmetry indicators corresponding to each of these 4-week periods. On the whole, it stands out that older adults having a negative asymmetry value tend to have more depressive symptoms than the other ones. This observation is confirmed for each AC and SC indicators by means of a Mann-Whitney comparison test under the null hypothesis {H_0_: GDS scores of older adults belonging to the *reactive* cluster are higher than those of others participants} which is not rejected with a p-value > 0.05. Interestingly, we also observe on the two figures that, while most of GDS scores strictly lower than 10 correspond to positive values of asymmetry, it also appears that quite a few positive values of asymmetry correspond to GDS scores higher than 10. It results from this particular situation that, by using a correlation analysis between GDS score and each of the asymmetry indicators, two significant (p-values < 0.05) but moderate negative Pearson’s rho are obtained: (1) −0.43 for AC, and (2) −0.40 for SC. Taken together, these results show that, while a *reactive* phone call activity tends to be associated with individuals having mild or severe depressive GDS scores, the *proactive* cluster contains both normal and depressive persons.

## Discussion

The use of the phone call direction parameter in CDRs analysis is still under-investigated in the current literature. In this paper, we propose to address this issue in a mental health context in a population of older adults. By using the concept of action-reaction in human behavior, we show that distinguishing outgoing phone calls and incoming phone calls leads to observe three different clusters in older adults, named (1) *proactive*, (2) *interactive*, and (3) *reactive*. We then introduce two asymmetry indicators which permit to synthetize these three phone call habits. We show the existence of a significant relation between each of these asymmetry indicators and the depressive health state of the older individual. Taken together, these results suggest the existence of relevant health-related insights contained in CDRs datasets when the phone call direction parameter is investigated.

Interestingly, on a psychological aspect, these results are in line with one of major assumptions of the behavioral theory of depression, as defined in the *Lewinsohn approach*, namely: “a low rate of response-contingent positive reinforcement acts as an eliciting stimulus for some depressive behaviors, such as feelings of dysphoria, fatigue, and other somatic symptoms^32^”, page 151). Under such an assumption, we could make two associations regarding phone call habits. On the one hand, social interactions through phone calls could be perceived as response-contingent, where response-contingent designates events experienced by a person that are made rewarding or unpleasant according to the nature of the response to a stimulus. On the other hand the existence of specific conditions in phone calls, as those with the restrain social network - friends and family -, specific discussions, or even the frequency of calls through the day, can define the positive/negative nature of the response to such a stimulus. In this train of thought, and based on our observations, it is plausible that positive values of asymmetry of phone call activity could be considered as a reflector of the concept of response-contingent positive reinforcement “*resconposre*”^32^ in older adults because of the negative correlation exhibited between asymmetry and GDS score. Also, complementary interpretations may be obtained by replacing this theory of reinforcement^32^ in the specific context of social psychology. Notably, we can make a parallel here between the asymmetry value calculated from phone call activity and the notion of social support that does represent a fundamental property of the older adult’s social environment^33,34^. There are actually both theoretical and practical evidences that this social property play a determinant role for mental health^28–31^, being associated with risky situations such as loneliness^29^ and social isolation^35^. In the present paper, these risky situations could potentially be reflected by a negative asymmetry value that highlights the fact that social solicitations originating from the older adult are lower than those received by him. This social discrepancy could be the sign of an intensification of the social support receiving by the older adult because of a particular situation alerting his close social network. Conversely, a positive asymmetry value could be the sign of a social environment’ solicitation decrease because of the absence of such an alert. Thus, as such, social asymmetry could be interpreted here according to two mechanisms (1) a reinforcement mechanism centered around the individual, and (2) a social support mechanism centered around the social environment. Taken together, the interest of the social asymmetry notion introduced in the present paper could reside (1) in permitting to model social interactions in accordance with these two mechanisms centered around two distinct axes – the individual and his social environment – but also (2) in proposing a social indicator measuring the balance between the social interactions occurring from these two axes, through respectively outgoing and incoming phone calls. Based on these assumptions, three main consequences stand out on a clinical aspect:

1. The present findings suggest that the phone device can be perceived as a social activity sensor whose passively generated data can reflect depressive symptoms of the older individual.
2. We provide two metrics that permit to assess the existence of such symptoms by measuring asymmetry in social interactions.
3. By using these two metrics calculated on passively collected data, we propose an automatic and non-invasive help for diagnosis tool that could be harnessed for earlier depressive symptoms detection in the older individual. This tool has the potential to provide him better and earlier guidance to the health practitioner based on objective social activity data measurements, and represents a complementary approach to subjective self-reports usually harnessed today^36^. Besides, because it is non-invasive and related to social interactions, such a tool could also be complementary with other innovative methods already existing in *mhealth*^37,38^, as with more classical approaches in Telemedecine, e.g., actigraphy which provides relevant results in physical activity monitoring of the individual but that dismisses other forms of activity as social interactions^39–43^.

However, to conclude on the significance of our results, a number of caveats and limitations have to be taken into account. Indeed, since we lead a descriptive analysis based on a relatively rich but small sample of 26 older individuals, any straightforward generalization of our results to the overall population might be avoided. Especially, since the proposed approach is, at the best of our knowledge, the first one that specifically investigates the phone call direction parameter by combining CDRs analysis with health data in older adults, whether and how similar results could be observed under different conditions, or in different sets of data, remain to be investigated. This could imply working on larger datasets, but also, more broadly, in leading analyses on different populations, such as young, disabled, or chronically ill individuals for instance.

Then, we have to insist on the fact that, although our results show the existence of a significant correlation between asymmetry in phone call activity and the depressive health state of the older individual, such a relation may not be necessary linear. Indeed, we observe in our sample that, among *proactive* users, quite a few obtain a GDS score under 10 whereas others obtain a GDS score corresponding to mild and severe depression. This implies two major points: (1) *proactive* activity in phone calls could possibly be divided itself into different sub-clusters containing both healthy and depressive individuals, and (2) depression could be found in both *reactive* and *proactive* clusters. Such insights could be in line with the co-existence of different forms of mental disorders in the individual, as anxiety and depression for instance^44,45^, and they could also explain, at least in part, the discrepancies in measures reported in recent studies^7^. Actually, it is plausible that individuals soliciting their social network more than responding to their solicitation could correspond both to healthy individuals as *social explorer* for instance^46^, but also to persons marked by a transitory emotional state as anxiety or suffering from a particular manic episode. Similarly, we can interpret that individuals having a prominent *reactive* attitude could correspond both to depressive persons, but also, simpler, to persons having a weak usage of the phone device or just to persons preferring to engage in conversation through other media supports as text messages or virtual social network for instance.

Consequently, as such, asymmetry in phone call activity, as other ‘objective’ passive measurements from phone datasets, should not have to be interpreted as an established and immutable health-related outcome rather a useful insight in *mHealth* which deserves to be included in multivariate analysis to offer more nuances in statistical analysis. Particularly, in health monitoring, such an inclusion could be valuable when combining machine learning models with the digital phenotyping concept^47,48^. Such an approach could permit, indeed, to enrich the ‘digital behavior’ of the individual using passive data of social interaction, which could potentially improve the performance of interpretable decision algorithms. In this train of thought, using machine learning models in *mHealth* by taking advantage of asymmetry indicators are included in our immediate plan. We believe that such an approach could provide an opportunity for assessing the relevance of the digital phenotyping concept^48^ for contributing to the future of mental health research and, more broadly, to investigate the interest of new technologies in improving health care monitoring.

## Methods

### Data collection and volunteers recruitment

Our dataset includes 12 months of CDRs of 26 older volunteers (20 women, 6 men; median age: 84 years; range: 71–91 years). CDRs were collected on their personal phone(s) and provided by their network communication operator. Each phone call detail record contains the date, hour, source used-ID, destination user-ID, direction and duration of the call (in seconds). Additionally, individuals having several phones registered by their network communication operator, for instance one or more landline phone(s) and/or one or more mobile phone(s), provided CDRs for all of them. Note that the identity of individuals and their phone contacts were anonymized. Then, in addition to CDRs datasets, participants passed a test every three months during the period of observation, with a health professional using, among others, the Geriatric Depression Scale (GDS) that assesses depression. The GDS is a 30-item questionnaire which is specifically designed for the depression assessment in elderly populations^49^. By calculating a GDS score based on the responses given to a binary (‘yes-no’) questionnaire, this scale permits to classify the older individual among three categories of subjects, namely (1) normal (score <10), (2) mildly depressed (9 < score < 20), and (3) severely depressed persons (score >19). Note that relying on GDS in depression assessment in older adults is motivated both by its frequent use in clinical practice, which is emphasized in the current literature^50^, as by the existence of a French and validated version of such a test^51^.

This study and the corresponding experimental protocols were approved by the French Commission for Data Protection and Liberties (CIL Register France Telecom 2011 n°44) at which the data collection phase of this project was originally initiated. All methods were performed in accordance with its regulations, written informed consent was obtained from all participants prior to data collection, and anonymization of participants’ data was applied ensuring privacy requirements.

### Data pre-processing step

As participants did not enroll the survey at the same time, and because the time of inclusion varies among them, we filter the CDRs dataset by selecting the time interval where most of the participants were enrolled in order to analyze CDRs extracted from the exactly similar time period. We then pre-process this CDRs dataset by selecting only participants who used their phone throughout the entire observation time period (12-month period) and who passed at least one health test. This procedure results on a set of 19 individuals as shown on Table 1. Additionally, note that only the incoming phone calls for which the individual responded were used in our analysis (i.e., dismissed incoming phone calls were removed).

**Table 1:** Call detail records dataset structure before and after data preprocessing.

	Before preprocessing	After preprocessing
Number of participants (according sex)	26 (F = 20, M = 6)	19 (F = 14, M = 5)
Age range [min; max] (years)	[71; 91]	[71; 91]
Averaged age (±standard-deviation) (years)	84 (±4)	84 (±5)
Total number of calls: outgoing; incoming	19198; 20062	17472; 17999
Number of calls by individual, 1st quartile: outgoing; incoming	285; 579	547; 634
Number of calls by individual, median: outgoing; incoming	590; 718	723; 903
Number of calls by individual, 3rd quartile: outgoing; incoming	944; 947	1140; 1100

**Table 2:** Descriptive statistics about the three phone call activity behaviors.

	All participants	Proactive participants	Interactive participants	Reactive participants
Number of participants	19	6	5	8
Monthly number of outgoing phone calls: Mean (SD)	77 (53)	135 (19)	68 (19)	42 (19)
Monthly number of incoming phone calls: Mean (SD)	79 (37)	78 (15)	65 (20)	88 (54)
Pearson’s r; p-value	0.27; 5.10e-05	0.53; 1.66e-06	0.74; 1.47e-6	0.62; 1.09e-11
Regression coefficient *: α; β	49; 0.35	45; 1.15	11; 0.80	19; 0.26
Mean of differences (limits of 95%-confidence interval)	−2 (−122–118)	57 (−46–159)	−2 (−37–33)	−47 (−138–45)

Descriptive statistics, correlation, regression, and Bland-Altman parameters for the monthly number of outgoing and incoming phone calls: all participants and by clusters. SD: standard deviation. *From the regression equation: *N*^*outgoing*^ = *α* + *β*. *N*^*incoming*^, where *N*^*outgoing*^ is the monthly number of outgoing phone calls variable and *N*^*incoming*^ is the monthly number of incoming phone calls variable.

### Asymmetry indicators

In order to characterize the individual’s phone call habit regarding the direction of his phone calls, we introduce two metrics measuring the balance between outgoing and incoming phone call activities, named (1) the asymmetry coefficient (AC), and (2) the skewness coefficient (SC).

### Asymmetry coefficient

We define the asymmetry coefficient, *AC*, between the number of outgoing phone calls and the number of incoming phone calls of one individual at a given time as:

$$AC(x,y)=\frac{out-in}{out+in}$$

where *out* represents the number of outgoing phone calls, and *in* represents the number of incoming phone calls.

Hence, the *AC* values vary between −1 and 1, where 1 is obtained when *in* is equal to zero (and *AC* tends to 1 when *in* becomes negligible with respect to *out*), where −1 is obtained when *out* is equal to zero (and *AC* tends to −1 when *out* becomes negligible with respect to *in*), and where 0 is obtained when *out* is equal to *in*. Thus, a high absolute value of *AC* reports a strong imbalance between the individual’s outgoing and incoming phone call activities, whereas a low absolute value reports more balance.

### Skewness coefficient

Similarly to *AC*, we define the skewness coefficient, *SC*, between the number of outgoing phone calls and the number of incoming phone calls of one individual at a given time as:

$$SC(x,y)=sign(out-in)\cdot (1-H(out,in))$$

where *out* represents the number of outgoing phone calls, *in* represents the number of incoming phone calls, and $H(.)$ is the Shannon entropy defined between *out* and *in* as: $H(out,in)=-(\frac{out}{out+in}{\log }_{2}(\frac{out}{out+in})+\,\frac{in}{out+in}{\log }_{2}(\frac{in}{out+in}))$. Similarly to *AC*, the *SC* values vary between −1 and 1, where 1 (respectively −1) is obtained when there is a total certitude to predict that, given one phone call of an individual and knowing his general phone call activity behavior, this phone call should correspond to an outgoing (respectively incoming) one. Inversely, a total incertitude corresponds to a *SC* value equal to zero.

### Data analysis

CDRs data are presented in the form of monthly, i.e., 4-weeks, number of phone calls over a total period of 12 months. Their analysis is divided in two successive processes, namely (1) the evaluation of balance in the older individual’s phone call activity regarding his outgoing and incoming phone calls, and (2) the investigation of a relation between such a balance and the depressive health state of the older individual.

### Evaluating asymmetry in phone call activity in older adults considering the phone call direction parameter

In order to investigate asymmetry in phone call activity in older adults regarding their outgoing and incoming phone calls, we distinguish individuals phoning more than responding to phone calls (*proactive* users), from those having an inverse behavior (*reactive* users). To this end, we compare the differences between the monthly number of outgoing phone calls and the monthly number of incoming ones, for each individual, using Wilcoxon signed-rank tests. These tests are based under the null hypothesis {H_0_: monthly number of outgoing phone calls ≥ monthly number of incoming phone calls}, which is rejected if the older individual has a r*eactive* behavior. In the case where H_0_ is not rejected, *proactive* users are then distinguished from those having an outgoing phone call activity similar to their incoming phone call activity (*interactive* users) by using Wilcoxon signed-rank tests under the null hypothesis {H_0_: monthly number of outgoing phone calls = monthly number of incoming phone calls}. We further analyze the association between the outgoing phone call activity and the incoming phone call activity by means of the Pearson’s correlation coefficient. Bland-Altman plots are then used to evaluate the agreement between both of these phone call activities. Last, in order to synthesize results about these three different phone behaviors, and to statistically confirm the existence of three distinct clusters, we introduce two asymmetry indicators characterizing the older individual’s asymmetry in his phone call activity regarding the phone call direction parameter. Hence, we verify that *proactive*, *interactive*, and *reactive* individuals originate from three different distributions using the Kruskal-Wallis comparisons test for each of these asymmetry indicators.

### Evaluating the relation between asymmetry in phone call activity and depression in older adults

In order to evaluate the relation between balance in phone call activity and depression in older adults, we measure the relation between both of the asymmetry indicators and the older individual’s GDS score by means of the Pearson’s correlation coefficient. The level of significance was set as p < 0.05 in all statistical tests. All statistical calculations were completed using the R software environment (version 3.1.0; R Foundation for Statistical Computing, Vienna, Austria).

## References

[1] Bhugra D (2017). The WPA- Lancet Psychiatry Commission on the Future of Psychiatry. Lancet Psychiatry.

[2] Decuyper, A. On the research for big data uses for public good purposes. Opportunities and challenges. *Netcom Réseaux Commun. Territ*. 305–314, 10.4000/netcom.2556 (2016).

[3] Saeb S (2015). Mobile Phone Sensor Correlates of Depressive Symptom Severity in Daily-Life Behavior: An Exploratory Study. J. Med. Internet Res..

[4] Bidargaddi N (2017). Digital footprints: facilitating large-scale environmental psychiatric research in naturalistic settings through data from everyday technologies. Mol. Psychiatry.

[5] Miller G (2012). The Smartphone Psychology Manifesto. Perspect. Psychol. Sci..

[6] the MQ Data Science group (2019). How data science can advance mental health research. Nat. Hum. Behav..

[7] Rohani DA, Faurholt-Jepsen M, Kessing LV, Bardram JE (2018). Correlations Between Objective Behavioral Features Collected From Mobile and Wearable Devices and Depressive Mood Symptoms in Patients With Affective Disorders: Systematic Review. JMIR MHealth UHealth.

[8] Jones KH, Daniels H, Heys S, Ford DV (2018). Challenges and Potential Opportunities of Mobile Phone Call Detail Records in Health Research: Review. JMIR MHealth UHealth.

[9] Blondel VD, Decuyper A, Krings G (2015). A survey of results on mobile phone datasets analysis. EPJ Data Sci..

[10] Palchykov, V., Mitrović, M., Jo, H.-H., Saramäki, J. & Pan, R. K. Inferring human mobility using communication patterns. *Sci. Rep*. **4** (2015). 10.1038/srep06174 PMC4141257 25146347

[11] Louail, T. *et al*. From mobile phone data to the spatial structure of cities. *Sci. Rep*. **4** (2015). 10.1038/srep05276 PMC4055889 24923248

[12] Mattie, H., Engø-Monsen, K., Ling, R. & Onnela, J.-P. Understanding tie strength in social networks using a local “bow tie” framework. *Sci. Rep*. **8** (2018). 10.1038/s41598-018-27290-8 PMC6008360 29921970

[13] Bengtsson, L. *et al*. Using Mobile Phone Data to Predict the Spatial Spread of Cholera. *Sci. Rep*. **5** (2015). 10.1038/srep08923 PMC4352843 25747871

[14] Monsivais, D., Bhattacharya, K., Ghosh, A., Dunbar, R. I. M. & Kaski, K. Seasonal and geographical impact on human resting periods. *Sci. Rep*. **7** (2017). 10.1038/s41598-017-11125-z PMC5587566 28878235

[15] Onnela J-P (2007). Structure and tie strengths in mobile communication networks. Proc. Natl. Acad. Sci..

[16] Weng Lilian, Karsai Márton, Perra Nicola, Menczer Filippo, Flammini Alessandro (2018). Attention on Weak Ties in Social and Communication Networks. Computational Social Sciences.

[17] Lambiotte R (2008). Geographical dispersal of mobile communication networks. Phys. Stat. Mech. Its Appl..

[18] Aledavood T, Lehmann S, Saramäki J (2018). Social network differences of chronotypes identified from mobile phone data. EPJ Data Sci..

[19] Faurholt-Jepsen M (2016). Behavioral activities collected through smartphones and the association with illness activity in bipolar disorder: Smartphone data in bipolar disorder. Int. J. Methods Psychiatr. Res..

[20] Barnett I (2018). Relapse prediction in schizophrenia through digital phenotyping: a pilot study. Neuropsychopharmacology.

[21] Varela, F. J., Thompson, E. & Rosch, E. *The embodied mind: cognitive science and human experience*. (MIT Press, 2000).

[22] Yerkes RM (1914). The Study Of Human Behavior. Science.

[23] Misawa Jimpei, Kondo Katsunori (2018). Social factors relating to depression among older people in Japan: analysis of longitudinal panel data from the AGES project. Aging & Mental Health.

[24] Aung MN (2016). The social network index and its relation to later-life depression among the elderly aged &ge;80 years in Northern Thailand. Clin. Interv. Aging.

[25] Keller BK, Magnuson TM, Cernin PA, Stoner JA, Potter JF (2003). The significance of social network in a geriatric assessment population. Aging Clin. Exp. Res..

[26] Cohen S (2004). Social Relationships and Health. Am. Psychol..

[27] Hupcey JE (1998). Clarifying the social support theory-research linkage. J. Adv. Nurs..

[28] Tough, H., Siegrist, J. & Fekete, C. Social relationships, mental health and wellbeing in physical disability: a systematic review. *BMC Public Health* **17** (2017). 10.1186/s12889-017-4308-6 PMC5422915 28482878

[29] Wang, J., Mann, F., Lloyd-Evans, B., Ma, R. & Johnson, S. Associations between loneliness and perceived social support and outcomes of mental health problems: a systematic review. *BMC Psychiatry* **18** (2018). 10.1186/s12888-018-1736-5 PMC5975705 29843662

[30] *Loneliness: a sourcebook of current theory, research, and therapy*. (Wiley, 1982).

[31] Schwarzbach M, Luppa M, Forstmeier S, König H-H, Riedel-Heller SG (2014). Social relations and depression in late life-A systematic review: Social relations and depression in late life. Int. J. Geriatr. Psychiatry.

[32] Lewinsohn, P. M. A behavioral approach to depression. In *The psychology of depression: Contemporary theory and research* xvii, 318–xvii, 318 (John Wiley & Sons, 1974).

[33] Seeman TE, Lusignolo TM, Albert M, Berkman L (2001). Social relationships, social support, and patterns of cognitive aging in healthy, high-functioning older adults: MacArthur Studies of Successful Aging. Health Psychol..

[34] Krause N (1986). Social Support, Stress, and Well-Being Among Older Adults. J. Gerontol..

[35] Wang J (2017). Social isolation in mental health: a conceptual and methodological review. Soc. Psychiatry Psychiatr. Epidemiol..

[36] Sano A (2018). Identifying Objective Physiological Markers and Modifiable Behaviors for Self-Reported Stress and Mental Health Status Using Wearable Sensors and Mobile Phones: Observational Study. J. Med. Internet Res..

[37] Lemola S, Perkinson-Gloor N, Brand S, Dewald-Kaufmann JF, Grob A (2015). Adolescents’ Electronic Media Use at Night, Sleep Disturbance, and Depressive Symptoms in the Smartphone. Age. J. Youth Adolesc..

[38] Kauer SD (2012). Self-monitoring Using Mobile Phones in the Early Stages of Adolescent Depression: Randomized Controlled Trial. J. Med. Internet Res..

[39] Ho, S., Mohtadi, A., Daud, K., Leonards, U. & Handy, T. C. Using smartphone accelerometry to assess the relationship between cognitive load and gait dynamics during outdoor walking. *Sci. Rep*. **9** (2019). 10.1038/s41598-019-39718-w PMC6395667 30816292

[40] Korszun A (2002). Use of actigraphy for monitoring sleep and activity levels in patients with fibromyalgia and depression. J. Psychosom. Res..

[41] Kim, H.-Y. *et al*. Monitoring for disease progression via mathematical time-series modeling: actigraphy-based monitoring patients with depressive disorder. In *Consumer Communications and Networking Conference (CCNC), 2013 IEEE* 56–61 (IEEE, 2013).

[42] Gershon A, Ram N, Johnson SL, Harvey AG, Zeitzer JM (2016). Daily Actigraphy Profiles Distinguish Depressive and Interepisode States in Bipolar Disorder. Clin. Psychol. Sci..

[43] Myers, D. R., Weiss, A., Rollins, M. R. & Lam, W. A. Towards remote assessment and screening of acute abdominal pain using only a smartphone with native accelerometers. *Sci. Rep*. **7** (2017). 10.1038/s41598-017-13076-x PMC5630621 28986551

[44] Goodwin, F. K., Jamison, K. R. & Ghaemi, S. N. *Manic-depressive illness: bipolar disorders and recurrent depression*. (Oxford University Press, 2007).

[45] Belmaker RH, Agam G (2008). Major Depressive Disorder. N. Engl. J. Med..

[46] Miritello, G., Lara, R., Cebrian, M. & Moro, E. Limited communication capacity unveils strategies for human interaction. *Sci. Rep*. **3** (2013). 10.1038/srep01950 PMC3674429 23739519

[47] Torous, J. *et al*. Characterizing the clinical relevance of digital phenotyping data quality with applications to a cohort with schizophrenia. *Npj Digit. Med*. **1** (2018). 10.1038/s41746-018-0022-8 PMC6550248 31304300

[48] Torous J, Onnela J-P, Keshavan M (2017). New dimensions and new tools to realize the potential of RDoC: digital phenotyping via smartphones and connected devices. Transl. Psychiatry.

[49] Yesavage JA (1982). Development and validation of a geriatric depression screening scale: A preliminary report. J. Psychiatr. Res..

[50] Michela B, Cataldi F, Carlucci L, Padulo C, Fairfield B (2018). Assessment of late-life depression via self-report measures: a review. Clin. Interv. Aging.

[51] Clément JP, Nassif RF, Léger JM, Marchan F (1997). Development and contribution to the validation of a brief French version of the Yesavage Geriatric Depression Scale. L’Encephale.

