# Author Correction: Association between social asymmetry and depression in older adults: A phone Call Detail Records analysis

**DOI:** 10.1038/s41598-020-57575-w

**Published:** 2020-01-14

**Authors:** Timothée Aubourg, Jacques Demongeot, Félix Renard, Hervé Provost, Nicolas Vuillerme

**Affiliations:** 1 Univ. Grenoble Alpes, AGEIS, Grenoble, France; 2 Orange Labs, Meylan, France; 3 LabCom Telecom4Health, Univ. Grenoble Alpes & Orange Labs, Grenoble, France; 4 Institut Universitaire de France, Paris, France

Correction to: *Scientific Reports* 10.1038/s41598-019-49723-8, published online 18 September 2019

This Article contains typographical errors in the methods section, where:

$$AC\,(x,y)=\frac{out-in}{out+in}$$

should read:

$$AC\,(out,in)=\frac{out-in}{out+in}$$

In addition,

$$SC(x,y)=sign(out-in)\cdot (1-H(out,in))$$

should read:

$$SC(out,in)=sign(out-in)\cdot (1-H(out,in))$$

Additionally, in Figures 4 and 5 the following annotation “Spearman’s rho = … (p.value < 0.05)” was incorrectly included.

The correct Figures 4 and 5 appear below as Figures 1 and 2 respectively.

**Figure 1:**
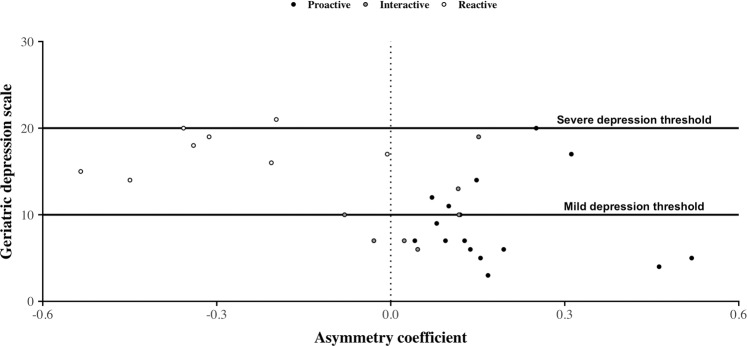
Geriatric Depression Scale values’ distribution according to asymmetry coefficient values. Here, dots correspond to the association between the individual’s GDS value and the individual’s asymmetry coefficient value calculated from CDRs over the 4-week period before the date at which the older adult passed the GDS test. Their colors are assigned according to the individual’s general phone call activity behavior. On this figure, we observe that individuals with a negative asymmetry coefficient tend to obtain a high GDS score corresponding mostly to mild depression. In contrast, individuals obtaining a low GDS score tend to have a positive asymmetry coefficient.

**Figure 2:**
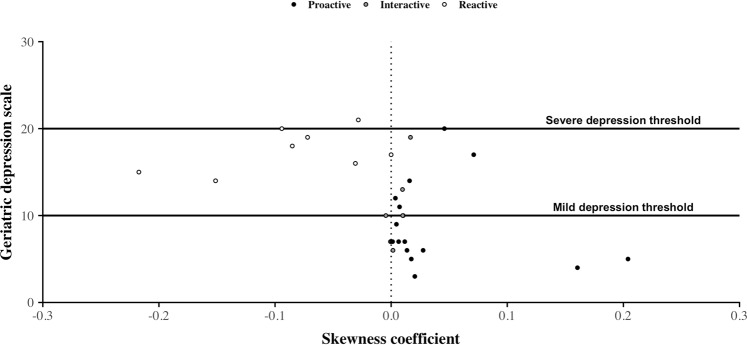
Geriatric Depression Scale values’ distribution according to the skewness coefficient values. Dots correspond to the association between the individual’s GDS value and the individual’s skewness coefficient value calculated from CDRs over the 4-week period before the date at which the older adult passed the GDS test. Their colors are assigned according to the individual’s general phone call activity behavior. On this figure, we observe that, similarly to the asymmetry coefficient, individuals with a negative value of their skewness coefficient tend to obtain a high GDS score corresponding mostly to mild depression. In contrast, individuals obtaining a low GDS score tend to have a positive value of information of direction.

